# Predictor of postoperative dyspnea for Pierre Robin Sequence infants

**DOI:** 10.1515/med-2020-0231

**Published:** 2020-09-30

**Authors:** Ning Yin, Lei Fang, Li Zhang, Yousong Cai, Guoxiang Fan, Xiaohua Shi, Hongqiang Huang

**Affiliations:** Department of Anesthesiology, Nanjing Medical University, Sir Run Run Hospital, Nanjing, China; Pneumology Clinic/Department of Biomedicine, University & University Hospital of Basel, Basel, Switzerland; Department of Anesthesiology, Children’s Hospital of Nanjing Medical University, 72 Guangzhou Road, Nanjing, Jiangsu, 210008, China

**Keywords:** Pierre Robin sequence, infants, postoperative dyspnea

## Abstract

The aim of this retrospective study is to determine the predictive factors of postoperative dyspnea in infants with Pierre Robin sequence (PRS). Forty children with PRS, who underwent general anesthesia, were retrospectively analyzed. The patient’s physiological status and anesthesiology data were collected accordingly, demographic characteristics including age, gender, height and weight at surgery, weight gain, preoperative airway status, tracheal intubation route, American Society of Anesthesiologists grading and airway Cormack–Lehane classification. Weight gain, dyspnea before the operation, Cormack–Lehane grade distribution showed a significant difference between patients with and without postoperative dyspnea (*p* = 0.0175, *p* = 0.0026, and *p* = 0.0038, respectively). Incompetent weight gain was identified as a predictor (*p* = 0.0371) of PRS postoperative dyspnea through the binary logistic regression model. In conclusion, this study established an early alerting model by monitoring the weight gain, dyspnea before the operation, Cormack–Lehane grade as potential combinations to predict the risk of postoperative dyspnea for PRS.

## 1 Introduction

The clinical triad consists of micrognathia (small mandible), glossoptosis (backward and downward displacement of the tongue), and airway obstruction is defined as Pierre Robin sequence (PRS) [1,2]. According to the previous literature review in 2015, PRS is a rare disease with an annual average of around 1:8,500 to 14,000 at births in neonates worldwide [3]. It is recognized that PRS is associated with intermittent upper airway obstruction, respiratory distress, and feeding difficulties, which put the affected infants at high risk within 1 month after birth [4,5]. Subsequently, the survived PRS infants may develop poor nutritional status, the inability of gaining weight, and slow growth. Patients with severe micrognathia and airway obstruction can be treated aggressively by mandibular distraction osteogenesis (MDO), which aims to correct the upper respiratory tract obstruction, to alleviate dysplasia and delay neurodevelopment caused by hypoxia [6,7]. However, such PRS infants often suffer from endotracheal intubation difficulties [8], poor hypoxia tolerance, and preoperative comorbidities, including pneumonia and heart failure [9]. All of these are great risks that anesthesiologists have to deal with [10]. The appropriate preoperative comprehensive score of PRS infants can help with difficult airway evaluation and optimized airway management strategy selection [11,12].

However, after the MDO operation, a few patients still have the risk of dyspnea during the perioperative period. Especially some of the low-weight immature infants with micro mandibular deformity have severe retrogression, retrogression, and narrow pharyngeal cavity. Although the mandible has been lengthened after operation, the pharyngeal cavity has not been enlarged sufficiently due to the short time after operation. The displaced tongue might continue to cause obstruction of the respiratory tract, and even the dyspnea might occur after operation. These postoperative situations are threatening patients’ lives, but how to identify these high-risk patients preoperatively has not been reported. There are not enough studies that predict the risk factors on postoperative dyspnea in PRS infants. To further investigate the potential solutions, we designed a retrospective study to analyze the collected patient’s key parameters with postoperative dyspnea. Thus, the objective of this study is to establish an optimized prediction model to identify predictive factors for postoperative dyspnea in children with PRS.

## 2 Materials and methods

### 2.1 Study subject’s recruitment

This study has been performed according to the Declaration of Helsinki, and the retrospective method performed in our study was approved by the local Independent Ethics Committee for Clinical Research of Children’s Hospital of Nanjing Medical University. The written informed consents have been collected from parents before conducting this study.

In this retrospective study of patient’s record, 40 PRS infants (23 males and 17 females) who underwent surgical treatment between 2007 and 2015 in Nanjing Children’s Hospital were recruited, and patient’s medical record and evaluation data were analyzed. The inclusion and exclusion criteria were described previously [2]: nine premature births and 31 full-term births infants, younger than 30 days after birth at the time of surgery, and the American Society of Anesthesiologists (ASA) classification category I–III were included. Patients with comorbidities that are not suitable for surgery were excluded. The following perioperative medical data of PRS infants were collected: age, sex, height, and weight at surgery (from the patient’s hospitalization record); preoperative airway status (evaluation record by anesthesiologist’s preoperative visit); and tracheal intubation route (recorded by surgical record). Nine premature delivered and 31 full-term normal delivered PRS infants were included in this study cohort (average body weight 2.68 ± 1.31 kg). All patients were younger than 30 days after birth at the time of surgery, among which 3 patients were younger than 20 days after birth, 13 were between 20 and 25 days after birth, and 24 were 26–30 days. The ASA classifications of all cases were within category I–III. The overall PRS preoperative breath and feeding difficulties were summarized in Table 1.

**Table 1:** Patient’s characteristics of preoperative breath and feeding difficulties (*n* = 40)

Characters	Signs and symptoms	Positive cases (%)
Upon breath	Chest retraction signs*	19 (47)
	Polypnea	6 (15)
Active body position	Side	21 (52)
	Prone	6 (15)
	Head low	3 (7)
Feeding difficulties	Feeding time extended	23 (65)
	Vomiting	12 (30)
	Nasogastric feeding	3 (7)

^*^Chest retraction signs: intercostal retractions, supraclavicular retractions, and/or suprasternal retractions.

### 2.2 Patient’s data collection

#### 2.2.1 Body weight gain

The patient’s body weight was measured by the digital baby weigh scale (Shanghai Guangzheng Medical Equipment Co., Ltd, DY-1digital baby weigh scale, No. 8845). The patient’s weight gain rate >20 g/day was recognized as growing satisfaction. Among the 40 patients, there were two patients who had a birth-to-surgery average daily weight gain of 10–20 g/day; 13 patients of 5–10 g/day; and 19 patients of <5 g/day. Overall, there were six patients whose weight was less than the birth weight at the date of surgery.

#### 2.2.2 Preoperative dyspnea

The patient’s oxygen saturation was measured by infant pulse oximeter (Philips Medizin Systeme Boeblingen GmbH, M8003A, No. 862115). Patients were slowly put into standard supine position from their autonomous free positions, while the SPO_2_ were recorded in both positions [13]. The results were evaluated as follows: 11 patients were considered to have inspiratory dyspnea (inhale three depressions sign, SPO_2_ 92–96%); 18 patients had breathing difficulties (SPO_2_ < 92%, supine position to lateral position SPO_2_ difference < 5%); six patients showed inspiratory dyspnea (SPO_2_ < 88%, supine position to lateral position SPO_2_ difference > 5%); and five patients had to undergo continuous positive airway pressure treatment.

#### 2.2.3 Cormack–Lehane classification

After anesthesia, the laryngoscope examination was performed to remark the infants’ Cormack–Lehane classification [14]: the completely visible glottis by the laryngoscope was considered as level 1 (one patient); only a glimpse of the glottic posterior joint was considered as level 2 (nine patients); only a glimpse of the epiglottis without appearance of glottis was considered as level 3 (22 patients); and unable to see any anatomical throat part was considered as level 4 (eight patients).

### 2.3 Statistical analysis

For the categorical variables, we showed the two-way classification table and performed Fisher’s exact tests to compare the differences of the distributions between two groups with/without postoperative dyspnea outcome. For the continuous variables, we showed the means and standard deviations. For variables with normal distribution and homogeneity of variance, we showed the means and standard deviations and performed *T* test to compare the difference, otherwise, using the Mann–Whitney *U* test. *P* value <0.05 was considered statistically significant. To build models using the demographic and clinic information to predict the postoperative outcomes, we fitted logistic regression model. All data analyses were performed using CRAN R (v3.4.1) and R packages rpart (Recursive Partitioning and Regression Trees) (v4.1-11) and pROC (1.10.0).

## 3 Results

### 3.1 Summary of demographic information and clinical data

As presented in Table 2, patients demographic information and clinical data showed that there is no significant difference of postoperative dyspnea between genders (*p* = 0.2973), ASA classifications (*p* = 0.1278), endotracheal intubation (*p* = 1.0), age (*p* = 0.262), and weight (*p* = 0.5829); however, preoperative dyspnea, Cormack–Lehane grades and weight gain significantly differentiated postoperative dyspnea (*p* = 0.0026, *p* = 0.0038, and *p* = 0.0175, respectively).

**Table 2:** Comparison of demographical and clinical data by outcome of postoperative dyspnea

Variable	Postoperative dyspnea (yes) (*n* = 11)	Postoperative dyspnea (no) (*n* = 29)	*p*-Value
Gender			0.2973
Female	3 (27.2%)	14 (48.3%)	
Male	8 (72.7%)	15 (51.7%)	
ASA			0.1278
I	5 (45.5%)	22 (75.9%)	
II	6 (54.5%)	7 (24.1%)	
Dyspnea before operation			0.0026
Yes	6 (54.5%)	2 (6.9%)	
No	5 (45.5%)	27 (93.1%)	
Cormack–Lehane grade			0.0038
I and II	2 (19.2%)	8 (27.6%)	
III	3 (27.2%)	19 (65.5%)	
IV	6 (54.5%)	2 (6.9%)	
Endotracheal intubation			1.0000
Yes	5 (45.5%)	12 (41.4%)	
No	6 (54.5%)	17 (58.6%)	
Age (day)	23.82 (±4.58)	25.59 (±3.34)	0.2620
Weight (g)	2625.46 (±424.72)	2705.86 (±346.51)	0.5829
Weight gain (g)	4.11 (±2.19)	6.42 (±3.42)	0.0175

Note: Data for variables such as gender, ASA, dyspnea before operation, Cormack–Lehane Grades, and endotracheal intubation were presented by patient number (percentages), while data for variables such as age, weight, and weight gain were presented by mean (±standard deviation). *P* value < 0.05 was considered statistically significant.

By analyzing patient’s demographic and clinical data, we could find out that (1) the distributions of gender, ASA grade, and endotracheal intubation were not significantly different between patients with and without postoperative dyspnea; (2) the mean values of age and weight were not significantly different between patients with and without postoperative dyspnea; (3) among all patients who had postoperative dyspnea, 6/11 = 54.55% had preoperative dyspnea, but this percentage was only 6.90% (*p*-value = 0.0026); (4) the higher Cormack–Lehane grades were significantly related to the increased risk of postoperative dyspnea (*p*-value = 0.0038); and (5) the mean weight gain of postoperative dyspnea and nonpostoperative dyspnea was 4.11 g (4.11 ± 2.19 g) versus 6.42 g (6.42 ± 3.42 g), with a significant difference (*p* = 0.0175). Therefore, the variables weight gain, dyspnea, and Cormack–Lehane grade might be predictors of the outcome of postoperative dyspnea.

### 3.2 Modeling and prediction of postoperative dyspnea

We fitted a binary logistic regression model by including all the variables listed in Table 3 and then chose the best fit using stepwise algorithm based on Akaike Information Criterion criteria. The following results demonstrate the fitted coefficient of the fitted logistic regression model to predict postoperative dyspnea.

**Table 3:** Logistic regression model to predict postoperative dyspnea

Variables	Estimate	Std. error	*z* value	Pr (>|*z*|)
Gender male	20.9069	5385.1994	0.004	0.9969
Age (day)	−0.4743	0.3029	−1.566	0.1174
Weight gain (g)	−1.0209	0.4898	−2.085	0.0371
Cormack–Lehane grade III	−0.1282	1.7524	−0.073	0.9417
Cormack–Lehane grade IV	45.0877	7826.1160	0.006	0.9954
Endotracheal intubation	21.6911	5678.7066	0.004	0.9970
(Intercept)	−25.5026	7826.1185	−0.003	0.9974

**Figure 1:**
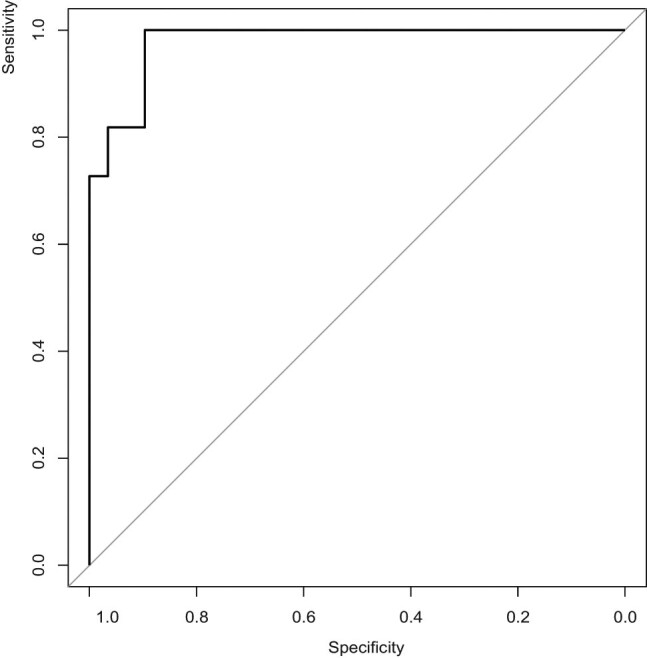
ROC curve of predicting postoperative dyspnea.

From Table 3, we could see that only weight gain was significant with *p*-value of 0.0371, while the others were not significant. This model could well predict the postoperative dyspnea with sensitivity of 100% and specificity 89.65%. The receiver operating characteristic (ROC) curve is shown in Figure 1. The area under the ROC curve was 0.9781. However, according to the data presented in Table 4, none of them could reach area under the curve (AUC) > 0.80. A simpler model including only two variables, dyspnea before operation and weight gain, could also produce good results with 81.82% sensitivity, 93.10% specificity, and AUC = 0.8918.

**Table 4:** Comparison of performances of single variables in predicting postoperative dyspnea

	Sensitivity	Specificity	AUC
Gender	0.727	0.483	0.605
Age (day)	0.909	0.414	0.619
ASA	0.545	0.759	0.652
Weight (g)	0.182	1.000	0.538
Weight gain (g)	0.727	0.862	0.754
Dyspnea before operation	0.545	0.931	0.738
Cormack–Lehane grade	0.545	0.931	0.760
Endotracheal intubation	0.455	0.586	0.520

From Table 4, we can observe that weight gain, dyspnea before operation, and Cormack–Lehane grade could achieve reasonably good performance to predict the postoperative dyspnea outcome.

## 4 Discussion

In this retrospective investigation, we analyzed preoperative age, gender, height and weight, ASA grade, dyspnea, and Cormack–Lehane grade of 40 neonatal PRS patients at the time of surgery, which aimed to predict postoperative complications. Weight gain, dyspnea before operation, and Cormack–Lehane grade could achieve reasonable performance to predict the postoperative dyspnea outcome independently. One previous investigation categorized PRS patients into three groups based on mortality [15]. MDO aimed to elongate the mandible. Bilateral osteotomies were performed, and a distraction device was applied for these infants [16]. Therefore, these patients could grow healthy. In our previous study, preoperative clinical manifestations (difficulties of breathing, body weight factors, and Cormack–Lehane grading scores) were collectively evaluated and quantified into a comprehensive scoring system to assess the patient´s intubation risk before surgery [2] and to predict the incidence of postoperative airway obstruction difficulties [2]. However, it did not predict postoperative dyspnea. Glossopexy (tongue–lip adhesion) procedure and MDO can improve PRS dyspnea and feeding problems [17,18,19], while the patient gains weight preoperatively. Mandibular advancement using MDO devices has been used in infants in an attempt to reduce the incidence of acute life-threatening airway obstruction [20].

The other investigators proposed a novel classification scheme that will better account for respiratory and feeding difficulties in PRS patients [21]. According to our clinical experiences as practice principles, the slower the PRS patients weight gain, the bigger degree the cleft palate might be indicated, which might accompany with higher chance of airway obstruction and feeding problems. However, the correlation of a high rate of velopharyngeal insufficiency after cleft palate repair in patients with PRS needs further investigation [22]. After operation, some patients might have a velopharyngeal problem and laryngeal development restriction, and some of them even have respiratory difficulties. Conflicting results and a lack of high-quality long-term prospective studies provided no conclusive evidence to compare the outcome benefit of cleft palate repair for PRS patients and cleft palate-only patients [23]. In our study, all 40 patients with PRS had underwent MDO, but some of them developed respiratory difficulties due to laryngeal development restricted after operation. In addition, some patients had preoperative weakness and gaunt very thin caused by bronchopulmonary dysplasia also presented as postoperative dyspnea. A resulting limitation of our research was that this disease had very low incidence, and the subject’s recruitment was limited; however, it was very much meaningful that we got this risk factor to predict postoperative dyspnea in 40 patients. Weight gain was a significant predictor. This might be because these patients had serious illness, they had a big cleft palate and small pharyngeal cavity, which led to difficulty in breathing, sucking, and feeding, and so they had difficulty to gaining weight [24]. Although these patients had undergone surgery, the pharyngeal cavity were still not enlarged, combined with poor preoperative nutrition and physical fitness, and they were more likely to have difficulty in breathing, as the resulting weight gain was related to postoperative dyspnea.

In conclusion, we conducted a logistic regression analysis to evaluate PRS postoperative dyspnea, which included patient’s clinical data including gender, age, weight gain, Cormack–Lehane Grade, and endotracheal intubation for consideration. According to the results, we have generated a model, using weight gain, dyspnea before the operation, and Cormack–Lehane grades potential predictors of postoperative dyspnea for PRS. The conclusion of this study emphasizes the importance and recommendation of monitoring PRS patient’s weight gain as a key measurement for clinical practice, and the further randomized prospective clinical study regarding this conclusion need to be conducted.
